# New Appliances and Things Medical

**Published:** 1910-10-15

**Authors:** 


### NEW APPLIANCES & THINGS MEDICAL.

**[We shall be glad to receive at our Office, 28 & 29 Southampton Street, Strand, London, W.C., from the manufacturers, specimens of all new preparations and appliances.]**

### TOOTH'S EXTRACT OF MEAT.

(12 Duke Street, London Bridge, S.E.)

We have received a sample of the above meat-extract, which is manufactured on the establishment of Robert Tooth, Esq., of Sydney. The extract of meat under consideration is useful for making beef-tea, soups, and gravies. For beef-tea half a teaspoonful of the extract dissolved in half a pint of water makes a stimulating and to some extent nourishing beverage of good and refreshing flavour. For the purpose of soup the extract can be used as a substitute for gravy beef, two teaspoonfuls or half an ounce of the extract being equivalent to one pound of gravy beef, or sufficient to make a quart of soup. The soup may be flavoured by the juice of fresh vegetables or by wine, and can be thickened if desired. Gravy can be made in precisely the same way, and may be thickened with flour and butter to taste. The extract is put up in small earthenware pots, keeps well when opened, and lends itself easily to the culinary manipulations mentioned above. We regard Tooth's Extract of Meat as being an exceedingly good article of its kind and likely to fill a very useful place in both domestic and invalid cookery.

### THE BRUSH "QUARTZLITE" LAMP

(The Brush Electrical Engineering Co., Ltd., Loughborough.)

One of the most interesting and remarkable inventions in connection with electric lighting is the "Quartzlite" lamp. Its burner consists of a tube of quartz containing a small quantity of mercury, through which the electric current is passed and manipulated by means of the ordinary tumbler switch. An intensely brilliant illumination is produced, with extraordinary powers of diffusion and penetration, showing up detail with exceptional sharpness. In its present stage the lamp produces the ultra-violet rays, which, although being distinctly destructive to bacterial life, detracts somewhat from its value for general purposes. When these rays are corrected and modified there is not the slightest doubt that the "Quartzlite" will find universal adoption, since it is the nearest approach to sunlight yet achieved in artificial lighting. One of the especial claims for this invention is its production of ozone, which is easily detected both by chemical tests and the sense of smell. This should render the lamp of considerable value in the lighting of large cities, as it will aid in the purification of the air and the destruction of disease germs, thus rendering town life more healthy and less devitalising than at present.

